# The impact of public health education on people's demand for commercial health insurance: Empirical evidence from China

**DOI:** 10.3389/fpubh.2022.1053932

**Published:** 2022-11-03

**Authors:** Lifei Gao, Ying Nie, Guojun Wang, Fei Li

**Affiliations:** ^1^School of Economics, Beijing Technology and Business University, Beijing, China; ^2^School of Insurance and Economics, University of International Business and Economics, Beijing, China; ^3^China Life Reinsurance Company Ltd., Beijing, China

**Keywords:** public health education, commercial health insurance, health literacy, health risk perception, health risk attitude

## Abstract

Public health education is gaining significance globally, and it is important for managing health risks. This study empirically analyzed the effect of public health education on people's demand for commercial health insurance. And we used the fixed effects and the mediating effect models, and instrumental variables regression in our research based on panel data of 31 provinces (including municipalities and autonomous regions) in China from year 2009 to 2019. The findings show that public health education significantly increases people's demand for commercial health insurance, and this effect remains significant when considering endogeneity and robustness. We further analyzed and found that the increased demand for commercial health insurance is caused by health literacy, health risk perceptions and health risk attitudes. Through heterogeneity analysis, we found that there were significant differences in the effects of public health education in regions with different demographic and socioeconomic characteristics. We found that the effect of health education on promoting people's demand for commercial health insurance is more obvious in regions with high levels of urbanization, proportion of men, education, economic development, medical resources, and social medical insurance coverage. Governments are supposed to take further measures to enhance the effectiveness of public health education, develop high-quality commercial health insurance, and continuously improve health risk coverage.

## Introduction

Health is an important form of human capital and the foundation of socioeconomic development (1). However, the current industrialized and information-oriented society contains risks, ecological and food safety problems. And the accelerating pace of life as well as work have significantly increased health risks. In recent years, the spectrum of human diseases is changing, and “civilization diseases” such as cardiovascular diseases, tumors, diabetes and mental disorders are gradually replacing infectious diseases as major threats to people's life and health. According to the latest statistics from the World Health Organization (WHO), 17.9 million people worldwide die from cardiovascular disease each year, accounting for 32% of all deaths (2). Over 422 million people suffer from diabetes around the world, and the number of cases and its prevalence are increasing every year (3). As to mental disorder, over 5% of adults worldwide suffer from the depression and more than 75% of people suffering from mental disorders in low- and middle-income countries do not receive treatment (4). According to latest research, there were 23.6 million new global cancer cases and 10 million cancer deaths in 2019 (5). However, people are often “overconfident” about health risks (6). They usually have an optimistic bias in their perception of health risks, believing that they are less susceptible to diseases than others (7, 8). They misperceive health risks and underestimate the impact of diseases on health (9, 10). Subjective perception of health risks may lead people to adopt health-hazardous behaviors or prevent them from taking effective health risk prevention measures (9). Therefore, they are reluctant to manage health risks. This leads to suppressed demand for commercial health insurance (6).

Public health education is a planned, organized, and systematic social education activity. Health education can effectively improve people's health literacy, thereby encouraging them to prevent and alleviate most “civilization diseases” by improving their living habits, strengthening physical exercise, and actively seeking medical care (11). Health education is essential for health awareness, disease prevention, and health promotion (12). Public health education can improve health risk perceptions (13), influence health risk attitudes, and increase health risk aversion (14). Theoretically, the improvement of health literacy, risk perception, and risk aversion can facilitate people taking measures to manage risks, such as diversifying health analysis by purchasing commercial insurance (15). Hence, public health education can promote active access to health care and increase the burden of health care expenditure, which would increase the demand for commercial health insurance. Generally, commercial health insurance is an effective tool for managing health risks. To the best of our knowledge, whether public health education encourages people to manage their health risks by purchasing commercial health insurance is an issue that remains unexplored.

As health risks to residents are increasing, the World Health Organization attaches great importance to health education and has held a global health promotion conference every 4 years since 1986, with health education as a core part of the conference (16). The Chinese government also highly values public health education. In terms of policy, China released the “Health China 2030” planning outline in 2016, which aims to strengthen health education and improve health literacy of the entire population. Regarding legislation, China introduced the Basic Medical Sanitation and Health Promotion Law in 2020, which clearly stipulates that “the state should establish a health education system, guarantee citizens' right to health education, and improve their health literacy.” In terms of professional institutions, China established the China Health Education Center in 2021, a professional institution dedicated to providing theoretical research and technical guidance for health education. According to the China Health Statistical Yearbook, by the end of 2020, China has held 792,300 public activities, provided training for 15,671,200 people both online and offline, and built 1,015 websites related to health education.

Years of public health education practices in China provide important support for our study. We focus on two core issues: (1) whether public health education significantly promotes health risk management by purchasing commercial insurance and (2) how public health education affects people's demand for commercial health insurance. This study uses provincial panel data from 2009 to 2019 to analyze the relationship between public health education and regional commercial health insurance development using a fixed effects model. Our findings suggest that public health education significantly increases the demand for commercial health insurance by improving health literacy, health risk perceptions, and changing health risk attitudes. This study makes three theoretical contributions: (1) we extend the literature by exploring the relationship between public health education and residents' demand for commercial insurance at the macro level; (2) we recommend further studies about the internal mechanism of public health education on residents' commercial insurance demand and unveil the “black box” between public health education and residents' health risk management behavior; (3) this study provides significant theoretical guidance for the government to strengthen health education, enhance the development of commercial health insurance, and improve residents' health risk protection.

The remainder of this manuscript is arranged as follows: The literature review section presents the theoretical background relevant to this study; the data, variables, and methods section describes the data source, variable selection, and model setting; the results section reports the basic regression results as well as endogeneity, robustness, mediating effects, and heterogeneity analysis; the discussion section includes discussion, limitations of the study and future outlook, along with research conclusions and policy implications.

## Literature review

### Public health education, health literacy, and demand for commercial health insurance

Public health education aims to improve residents' health literacy (17), encourage people to change bad habits, and actively seek medical care. It is effective in increasing health literacy (12, 18), changing hygiene habits (19, 20), improving personal hygiene for protection from infectious diseases, and further reducing the risk of acute illness (21). Seeking medical treatment is an effective measure to treat diseases and improve health at the cost of increasing medical expenses. Despite the existence of some social security systems in countries around the world, the level of healthcare coverage remains limited and residents face the burden of health costs, which greatly reduces access to health care (22). Health education enables people to recognize the significance of health, promote their active access to healthcare, and seek ways to relieve pressure on healthcare spending (11). Commercial health insurance is an effective way to manage health risk. As an effective supplement to social medical insurance, commercial health insurance can effectively relieve the pressure on residents' medical expenses by paying out insurance benefits and reimbursing medical expenses (23). Previous studies have found that residents' willingness to purchase commercial health insurance significantly increases when there is increased healthcare spending expected in the future (24, 25).

### Public health education, health risk perception, and demand for commercial health insurance

Health risk perception is essential for managing health risks. It reflects how an individual perceives and recognizes the possibility and impact of health risks based on their knowledge and experience (26), that is essential for managing health risks. However, people usually have inadequate knowledge about health risks (7, 27), which hinders people from effectively managing health risks. Public health education remedies these tendencies (28), and improves health risk perception (29, 30). It also informs the public about the probability and severity of diseases, enabling timely risk identification and enhanced health-risk management (13, 30). In terms of public health education, targeted and personalized public health education can be more effective in improving health risk perception (18). Owing to the impact of the Corona Virus Disease 2019 pandemic (COVID-19), people worldwide have received extensive health education related to COVID-19. The level of risk perception regarding the COVID-19 pandemic has significantly increased due to public health education, and people are managing their own health risks by wearing masks and washing their hands regularly (15, 31). Commercial health insurance is an important tool for health risk management. Through health education, people's health risk awareness level is enhanced, which in turn promotes residents to strengthen health risk management, thereby enhancing their willingness to purchase commercial health insurance (23, 32). Previous studies have found that residents are more likely to purchase commercial health insurance when they are fully aware of the health effects of haze (23). Low perception of long-term care risks predominantly causes the low demand for long-term care insurance in many countries (33). There is a significant increase in residents' willingness to purchase long-term care insurance after being informed of the probability of long-term care risks and cost of care (34). Adequate health risk communication can increase the level of health risk perception of the population, which consequently promotes the purchase of commercial health insurance (35).

### Public health education, health risk attitudes, and demand for commercial health insurance

Health risk attitudes are highly correlated with an individual's overall risk appetite, reflecting their aversion to health risks related to certain behaviors, and have an important influence on the demand for commercial health insurance (26). The occurrence of events such as severe weather, natural disasters, and epidemics can increase the risk aversion of the population (36, 37). Health education has a similar effect; for example, ongoing health education about the dangers of mad cow disease may increase risk aversion and even make residents pay higher prices to ensure food safety (14). Owing to the global spread of the COVID-19 pandemic, countries have intensified health education on epidemic protection for their populations, and their risk aversion has considerably increased (31, 36). According to the insurance demand theory, risk-averse people prefer to obtain protection by purchasing insurance (38, 39); the higher the risk aversion, the stronger the residents' willingness to purchase (40). The fact that a deepening risk-averse attitude promotes insurance has been proven for various types of insurance such as earthquake insurance (37, 41), technology insurance (42), agricultural insurance (43), social health insurance (44), and commercial health insurance (28, 39).

**Figure 1:**
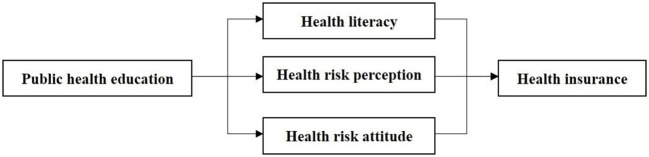
Theoretical framework.

According to the literature, public health education can improve residents' health literacy and encourage people to actively seek medical care to improve their health. The increasing pressure on medical expenditures tends to encourage individuals to reduce financial risks by purchasing commercial health insurance. As public health education enhances health risk perception and reduces health risk perception bias, individuals who have adequate health risk perception may manage their health risks by purchasing commercial health insurance. Additionally, public health education affects people's health risk attitude and further accelerates the purchase of commercial health insurance. Theoretically, public health education can encourage residents to purchase commercial health insurance through health literacy, health risk perception and health risk attitudes (Figure 1). Although there are some studies on the relationship between healthcare expenditures, health risk perception, health risk attitudes, and demand for commercial health insurance, few studies have focused on the relationship between public health education and residents' demand for commercial health insurance.

## Materials and methods

### Data sources

We constructed provincial panel data based on public data, which covered information on 31 provincial administrative regions in China from 2009 to 2019. The data on commercial health insurance, the data on public health education, and the rest of the data are from the Yearbook of China's Insurance (45), the China Health Statistical Yearbook (46) and the China Statistical Yearbook (47), respectively.

### Variables

#### Explanatory variable

The core explanatory variable was public health education. According to previous studies, the most effective methods of public health education are face-to-face public health education and Internet public health education (11). They also suggest that the use of the Internet can effectively promote the purchase of commercial health insurance (48). A comprehensive health education score (Public health education) was constructed using the entropy value method to measure public health education in each region. Two offline health education indicators (public health education activities and training attendance) and one online health education indicator (health education websites), were used to develop a composite indicator for public health education. Considered as an objective assignment method, the entropy method can effectively overcome the shortcomings of information superposition among variables and the subjectivity of artificially determined weights and is now widely used in socioeconomic research. Referring to existing studies, our study uses the entropy value method to obtain the weights and calculates the status of public health education in 31 provinces (including autonomous regions and municipalities) of China from 2009 to 2019 based on these weights, as detailed in Table 1.

**Table 1:** Comprehensive evaluation index system for health education.

**Index**	**Comentropy**	**Redundancy**	**Weight**
Health education activities	0.946	0.054	0.454
Health education Websites	0.938	0.062	0.521
Health education activities attendance	0.997	0.003	0.025

#### Explained variable

The variable explained in our study is the demand for commercial health insurance. We used the commercial health insurance density indicator which shows per capita commercial health insurance premium expenditure to measure the demand for commercial health insurance among regional residents. Compared to regional commercial health insurance premium income, commercial health insurance density indicators are more comparable across regions (49). Additionally, to prevent non-stationary data from affecting the results, we used the logarithm of commercial health insurance density to measure the demand for commercial health insurance.

#### Control variables

According to previous studies, economic development (50, 51), medical resources (52–54), education (55–57), urbanization (51, 58), population dependency (59), health (24), gender (53, 57), social health insurance participation (20, 60), and air pollution may influence people's demand for commercial health insurance. We control for these factors in our study to ensure the reliability of our findings. Economic development is measured as the logarithm of GDP per capita. The medical resources are measured considering the number of staff of medical and health institutions per 1,000 people. The percentage of the population with a college diploma or above is used to indicate the education level of the region (Education). Urbanization ratio was calculated as the proportion of the urban resident population to the total resident population. The dependency ratio is measured by the ratio of the population aged 65 and over and 14 and below to the resident population. We used the mortality rate as a proxy for the health status of the regional population. The gender ratio of the resident population was used to represent the gender profile of the study population. The proportion of the population covered by social health insurance to the total population was used to express the regional level of social health insurance coverage (23). The regional air pollution status is indicated by industrial sulfur dioxide emissions.

### Methods

This study examines the effect of public health education on demand for commercial health insurance at the macro level. Considering the interference of unobservable factors in each province and special policy releases in particular years on the empirical results, we use a panel fixed-effects model to control for features that do not vary with individuals and time. Therefore, we constructed the following model:


$$\begin{array}{l} lndensity_{it}=\alpha +\beta {\left(health\;education\right)}_{it}+\gamma \;\cdot \;control_{it}\\ \;+\mu_{i}+{\text{λ}}_{t}+\varepsilon_{it} \end{array}$$

where *lndensity*_*it*_ denotes the natural logarithm of health insurance density in province i in year t, (*health education*)_*it*_ denotes the level of public health education in province i in year t, *control*_*it*_ denotes a series of control variables, μ_*i*_and λ_*t*_ represent individual effects and time effects, and ε_*it*_ is a random disturbance term. Moreover, the Hausman test results show that it is appropriate to use a fixed effects model in this study.

### Statistical analysis

#### Descriptive statistical analysis

Descriptive statistics were used to demonstrate the statistical characteristics of the explained, explanatory, and control variables. The results of the descriptive statistics for each variable are presented in detail in Table 2.

**Table 2:** Descriptive statistics of variables.

**Variable**		**Mean**	**Standard deviation**	**Min**	**Max**
Explained variable	Health insurance density (RMB per person)	4.66	1.09	1.28	7.51
Explanatory variable	Public health education	0.24	0.17	0.01	0.77
Control variables	Economic development (RMB per person)	10.69	0.50	9.24	12.01
	Urbanization ratio (%)	56.09	13.75	22.3	89.6
	Gender ratio (%)	104.89	3.99	95.77	123.17
	Health Status (%)	6.02	0.78	4.21	7.57
	Dependency ratio (%)	36.41	6.67	19.27	51.45
	Education (%)	12.79	7.21	1.68	50.49
	Social health insurance coverage (%)	0.54	0.30	0.12	1.11
	Medical resources (per 1,000 persons)	5.83	1.79	2.37	15.46
	Air pollution (tons)	437,944	363,875	880	1,628,647

#### Spatio-temporal evolution analysis

**Figure 2:**
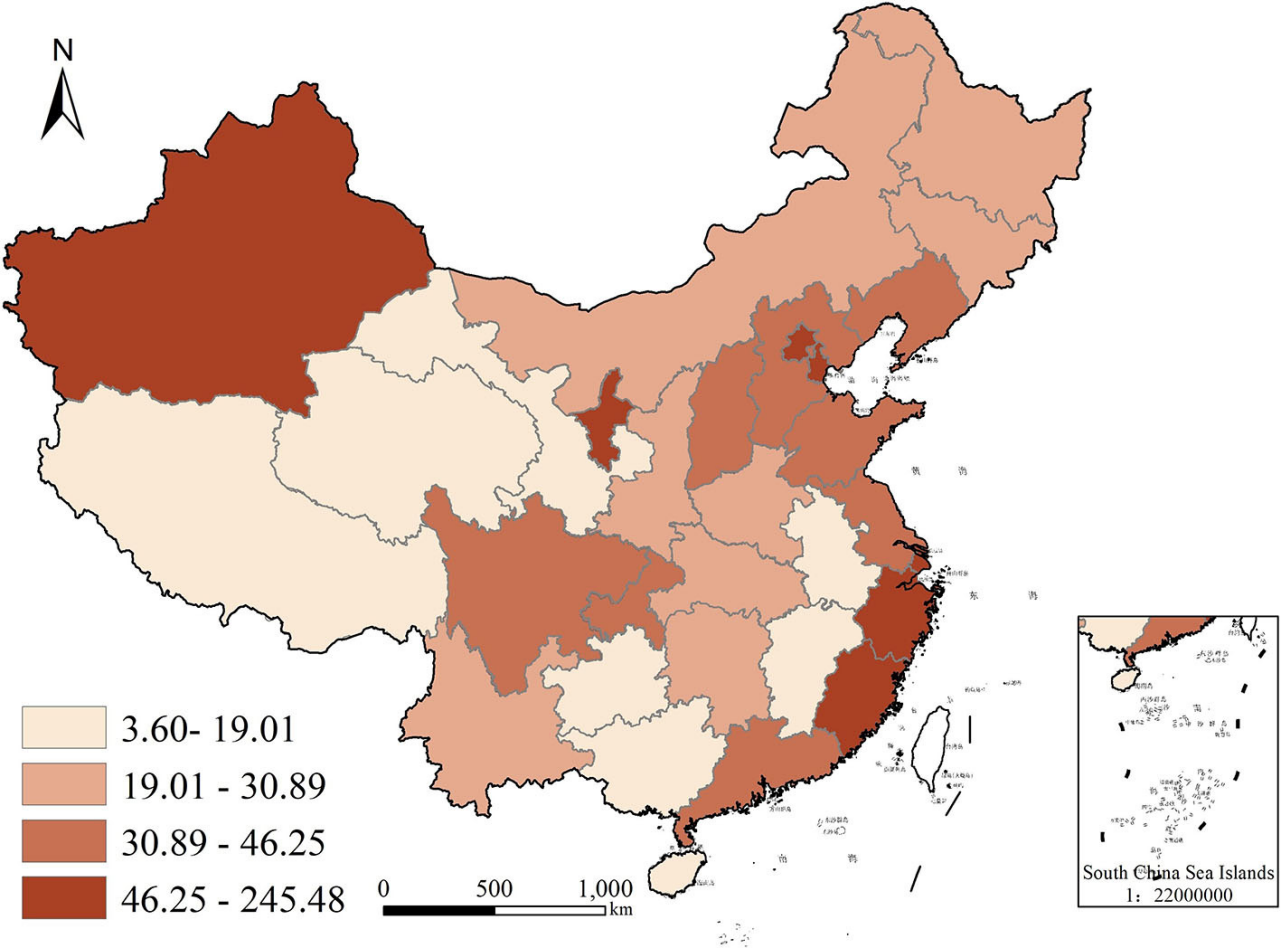
Commercial health insurance density in China in 2009.

**Figure 3:**
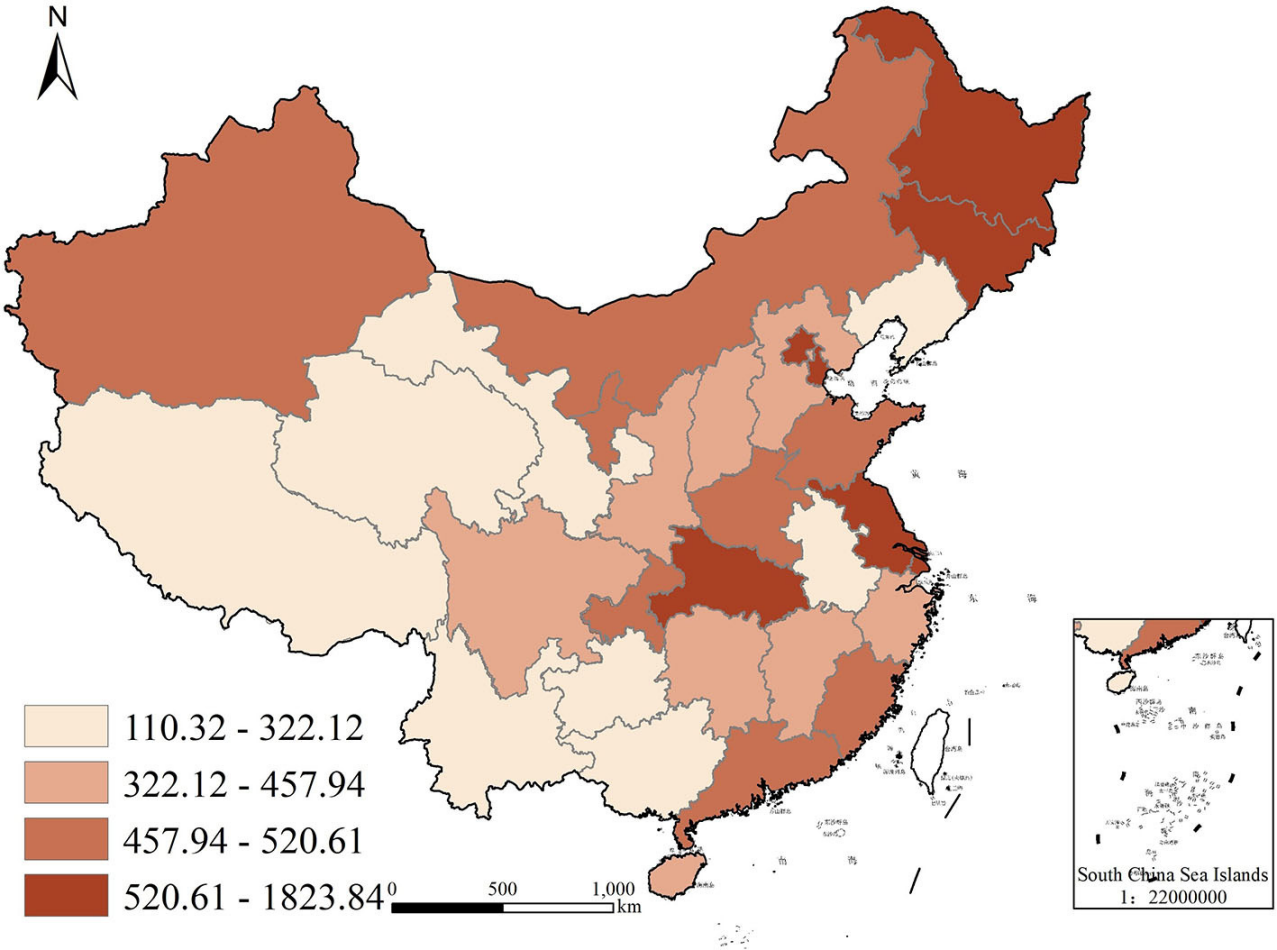
Commercial health insurance density in China in 2019.

**Figure 4:**
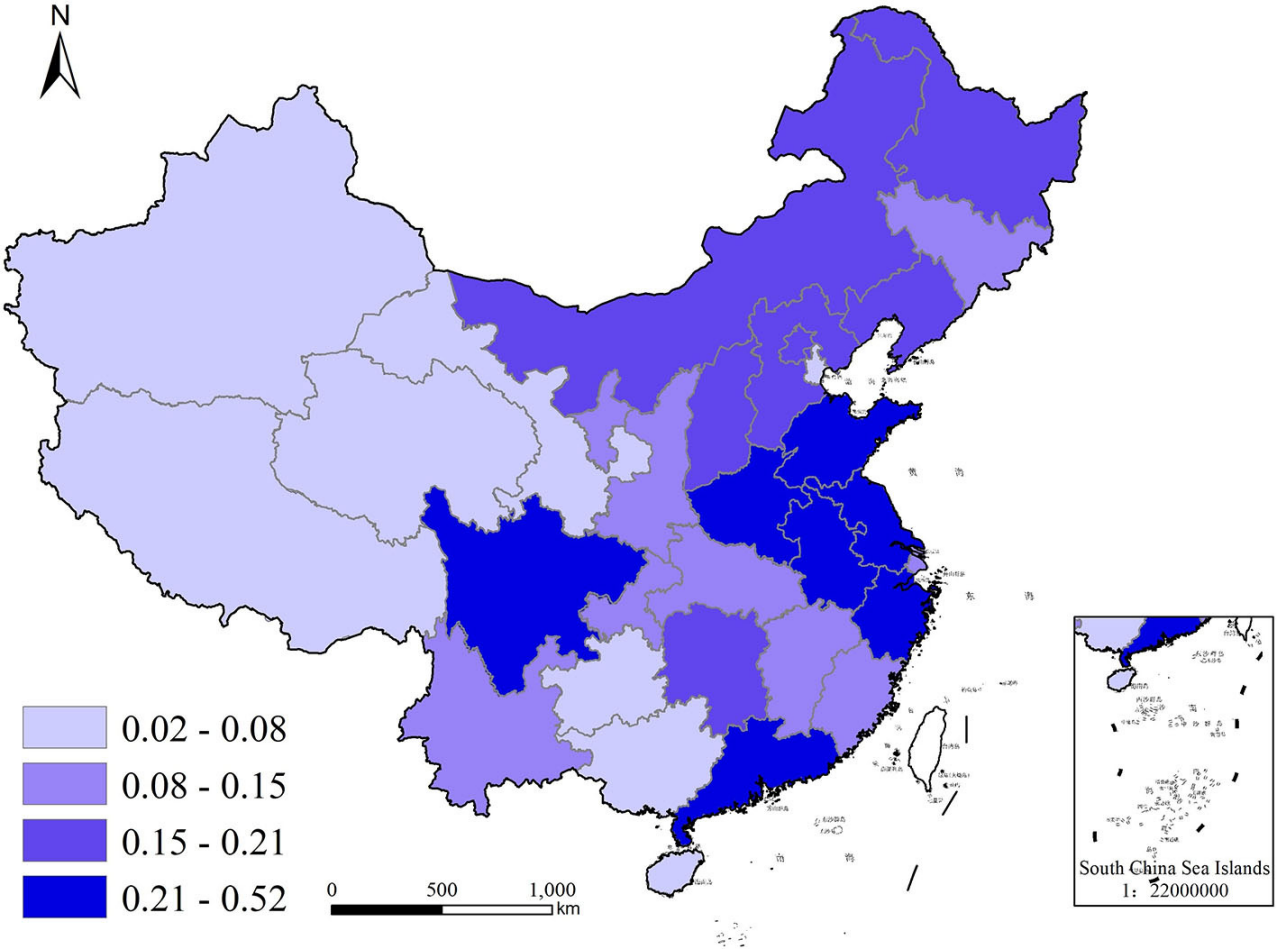
Public health education in China in 2009.

**Figure 5:**
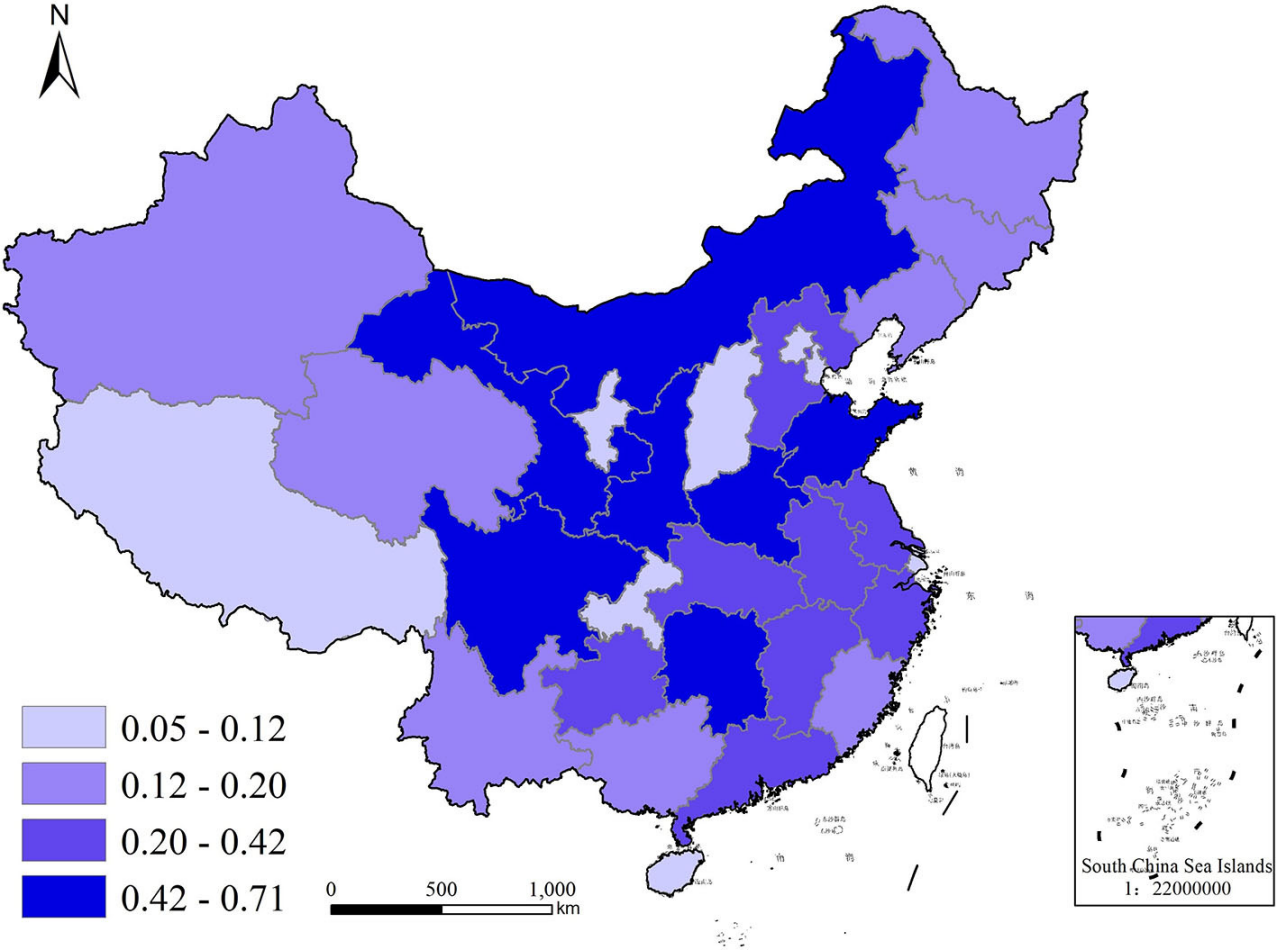
Public health education in China in 2019.

To better visualize the demand for commercial health insurance and public health education in each region of China, we used ArcGIS10.5, a geographic analysis software with version 10.5, to plot quadrature chart of health insurance density and public health education in 2009 and 2019, as shown in Figures 2–5. In terms of health insurance density, there was a significant increase in all regions in 2019 compared with 2009. Figures 2, 3 show that the provinces with higher demand for health insurance are mainly located in the eastern coastal region such as Beijing, Shanghai and Jiangsu, and the level of demand for commercial health insurance in the western region is generally lower. In terms of the level of public health education, after 10 years of development, the level of public health education in the central and western regions improved significantly. According to Figure 5, the overall level of public health education is now more balanced across the nation in 2019. According to Figures 2–5, provinces with high health insurance density tend to have higher levels of health education, which is somehow consistent with our expectations.

#### Panel regression analysis

We studied the effect of public health education on residents' demand for commercial health insurance using panel fixed-effects model, and the regression results are presented in Table 3. Column (1) shows the regression results without control variables, where public health education is significant at the 1% level. Column (2) shows the regression results after adding control variables. The effect of public health education is still significant, and the goodness of fit is above 0.9 in all cases, which indicates that the model is well fitted. The above regression results indicate that public health education can effectively increase residents' demand for commercial health insurance. For every single unit increase in the level of public health education, the density of commercial health insurance is increased by 34.84%.

**Table 3:** The impact of public health education on the demand for commercial health insurance.

**Variables**	**Health insurance density**		**Public health education**	**Health insurance density**
	**(1)**	**(2)**	**(3)**	**(4)**
			**First stage**	**Second stage**
Public health education	0.494*** (2.90)	0.348* (1.96)		1.383* (1.72)
IV			0.822*** (3.96)	
Economic development		0.109 (0.62)	−0.040 (−0.71)	0.160 (0.87)
Urbanization ratio		−0.002 (−0.14)	0.006 (1.24)	−0.014 (−0.83)
Gender ratio		−0.003 (−0.59)	−0.001 (−0.59)	−0.001 (−0.14)
Dependency ratio		−0.003 (−0.40)	0.002 (0.71)	−0.003 (−0.41)
Health status		0.069 (1.44)	−0.009 (−0.61)	0.079 (1.59)
Education		−0.020** (−2.15)	0.001 (0.18)	−0.021** (−2.16)
Social health insurance coverage		0.115 (1.14)	−0.215 (−0.66)	0.120 (1.15)
Medical resources		0.008 (0.30)	0.012 (1.35)	−0.005 (−0.16)
Air pollution		−0.000* (−1.90)	−0.000** (−2.00)	−0.000 (−1.37)
Time effect	Yes	Yes	Yes	Yes
Individual effect	Yes	Yes	Yes	Yes
observations	341	341	341	341
R-squared	0.938	0.941	-	-

^*^p < 0.1, ^**^ p < 0.05, ^***^ p < 0.01; Standard errors are shown in parentheses.

#### Endogeneity analysis

The relationship between public health education and demand for commercial health insurance is influenced by multiple factors. Although we has taken the economy, population, medical resources, education level, and other factors into consideration, there may be endogeneity problems in the model, such as omitted variables. To overcome the endogeneity issues, we used public health education at the regional level as an instrumental variable for public health education at the provincial level. Referring to previous studies (61), the former is highly correlated with the latter, and it is not directly related to the demand for health insurance (62, 63). According to the criteria of the China Health Statistical Yearbook, we classify the 31 provincial administrative regions into three regions: east, middle, and west. Public health education on the three regional levels is used as an instrumental variable in a two-stage least squares regression.

Columns (3) and (4) in Table 3 report the results of the first- and second-stage regressions. The coefficient for public health education at the regional level in the first-stage regression results was 0.822 and significant at the 1% level. Moreover, the F-statistic in the one-stage regression was 15.691, which was > 10. According to the relevant criterion (64), weak instrumental variable hypothesis is rejected, which indicates that the instrumental variable is valid. The regression results of the second stage show that the coefficient of public health education level remains significantly positive, indicating that our results are reliable.

#### Robustness analysis

We used three methods to test robustness of our results: replacing the explained variable, changing the explanatory variable, and deleting data for the year of significant policy implementation. And panel fixed effect models were used to test robustness.

##### Replacing explained variable

Although commercial health insurance premium income cannot measure commercial health insurance demand at the individual level as insurance density does, it considerably reflects the level of commercial health insurance demand of the total population in a region. Therefore, we used commercial health insurance premium income as the explanatory variable to test the robustness of our model. Column (1) of Table 4 shows the regression results, which indicate that the effect of public health education remains significantly positive.

**Table 4:** Robust test results.

**Variables**	**Premium**	**Density**	
	**(1)**	**(2)**	**(3)**
Health education	0.297*		0.404**
	(1.70)		(2.04)
Health education dummy		0.090*	
		(1.88)	
Time effect	Yes	Yes	Yes
Individual effect	Yes	Yes	Yes
Observations	341	341	310
R-squared	0.946	0.941	0.943

^*^ p < 0.1, ^**^ p < 0.05, ^***^ p < 0.01; Standard errors are shown in parentheses.

##### Replacing explanatory variable

Furthermore, we constructed a binary variable to indicate public health education. When a province's health education in a year is greater than or equal to the median value of the provinces in that year, the value is assigned as 1, otherwise, it is assigned as 0. As presented in Column (2) of Table 4, public health education is statistically significant, which means that our findings are robust.

##### Deleting data in special year

The implementation of significant policies affects the development of the industry and people's behavior. To exclude the effect of policy implementation, we sorted through the commercial health insurance policies during the sample period. We found that “Several Opinions on Accelerating the Development of Commercial Health Insurance” was issued by the state council in October 2014, specifically addressing the development of the commercial health insurance industry, and it has promoted the development of commercial health insurance in China. There is a lag in the policy's effect since it was released at the end of the year. Hence, we regarded 2015 as the year of the policy shock and estimated the basic model after dropping data in 2015. Column (3) of Table 4 shows that after deleting the data on the special year, the effect of public health education is still significant.

The results of the three robustness tests indicate that the findings of this study are robust. Hence, the conclusion that public health education can increase people's demand for commercial health insurance is reliable.

#### Mechanism analysis

Public health education significantly increases people's demand for commercial health insurance, however, what is the mechanism of this influence? The mediating effect model can help us to find out the answer. Mediating effect model has been widely used as a primary tool for testing this mechanism (65). Bootstrap testing is an increasingly popular method for testing mediating effects. The bootstrap method, as a nonparametric repeated sampling method, has no strict restrictions on the distribution of the mediating variables. Compared to the Sobel test, it can be applied in cases where the samples are not normally distributed, making the research results more reliable. To answer the above question, we used the bootstrap method to test the mediating effects of three indicators: health literacy, health risk perception, and health risk attitudes. Table 5 reports the results of the mediating-effects test.

**Table 5:** The result of the mediating effect model.

**Mediating variable**		**Effect**	**Coefficient**	**Standard error**	**95% confidence interval**	
					**Lower limit**	**Upper limit**
Health literacy	Outpatient visits	Indirect	0.071**	(0.03)	0.007	0.136
		Direct	0.384***	(0.11)	0.171	0.597
Health risk perception	Physical examination	Indirect	0.131**	(0.07)	0.002	0.260
		Direct	0.334***	(0.13)	0.080	0.589
	Smog Baidu index	Indirect	0.122***	(0.05)	0.030	0.214
		Direct	0.343***	(0.12)	0.116	0.570

^*^ p < 0.1, ^**^ p < 0.05, ^***^ p < 0.01; Standard errors are shown in parentheses.

##### Health literacy

According to the theoretical analysis, public health education can effectively improve people's health literacy making them actively seeking medical treatment (11, 66). Hence, people's pressure on medical expenditure increases, which may encourage them to transfer their medical expenses to insurance companies through commercial health insurance. Therefore, to explore whether public health education increases the demand for commercial health insurance through health literacy, we obtained the average outpatient visits of residents from the China Health Statistical Yearbook as a proxy variable for health literacy. According to the regression results in Table 5, both the direct and indirect effects of health literacy were significantly positive, and the 95% confidence interval did not contain zero. The results demonstrate that health literacy plays a partially mediating role.

##### Health risk perception

Public health education can increase residents' health risk perceptions, which encourages them to better manage health risks using tools such as commercial health insurance (28, 32). Hence, the higher the perception of health risks, the more people can recognize the dangers and importance of health risks. Residents identify their health risks through medical checkups. Therefore, we obtain the physical examination data of residents in each province from the China Health Statistics Yearbook as a proxy for health risk perception. The long-standing haze problem in China is a serious threat to human health. People with higher levels of health risk perception would be more concerned about haze and better understand its hazards and protective measures. Baidu is the largest search engine in China, people usually search for relevant information from Baidu; thus, the Baidu index can truly reflect people's attention to a certain topic. Hence, we obtained the Baidu index for the term “haze” from its website (https://index.baidu.com) and used it as another proxy for health risk perception. According to the results in Table 5, the direct and indirect effects of both proxies are significantly positive, and zero is not included in the 95% confidence interval. These results indicate that health risk perception plays a partially mediating role.

##### Health risk attitudes

Public health education can change people's attitude toward health risks, making them more averse to health risks, thereby, promoting the purchase of commercial health insurance (14, 28). Individual health behaviors have been commonly used in previous studies to represent individual risk attitude (39). There is a global consensus that smoking is hazardous to health and that the act of quitting can represent a change in one's attitude toward health risks. Generally, it is difficult to quit smoking, and it requires the help of certain methods. People who intend to quit smoking often search for knowledge and methods about it. Therefore, we obtained the Baidu index for “quit smoking” from its website as a proxy variable for health risk attitudes. According to the regression results in Table 5, both the direct and indirect effects of the Baidu index of smoking cessation were significantly positive, and the 95% confidence interval did not contain zero, indicating that health risk attitudes played a partially mediating role.

#### Heterogeneity analysis

Given that the effects of the demand for commercial health insurance is influenced by demographic and socioeconomic characteristics, including urbanization ratio, gender ratio, education level, economic development, medical resources, and social medical insurance coverage, the research samples were further divided into subgroups based on these six variables. The panel fixed effects models were conducted, and the results were displayed in Table 6. Overall, in terms of demographic characteristics, public health education in areas with high levels of urbanization, high proportion of men, and high levels of education is more likely to significantly increase people's demand for commercial health insurance. Regarding economic and social development, public health education may significantly increase the demand for commercial health insurance among people in areas with high economic development level, abundant medical resources, and high social medical insurance coverage.

**Table 6:** Heterogeneity analysis of the impact of health education on health insurance.

**Variables**	**Gender ratio**		**Education**		**Urbanization ratio**	
	**High**	**Low**	**High**	**Low**	**High**	**Low**
Health education	0.519***	0.280	0.602***	0.068	0.570***	0.089
	(3.00)	(0.84)	(3.26)	(0.21)	(3.27)	(0.25)
**Variables**	**Economic development**		**Medical resources**		**Social medical**
					**insurance coverage**	
	**High**	**Low**	**High**	**Low**	**High**	**Low**
Health education	0.309**	0.167	0.496***	0.173	0.548***	0.165
	(2.13)	(0.50)	(3.08)	(0.48)	(2.86)	(0.48)

^*^p < 0.1, ^**^ p < 0.05, ^***^ p < 0.01; Standard errors are shown in parentheses.

## Discussion

In the contemporary society, people are exposed to a wide range of risks, especially increasing health risks that pose a great threat to human health and life (67). Despite this critical situation, people tend to under-perceive health risks and are overoptimistic about it (68), which is not conducive to scientific and effective health risk management. Health risks challenge the stable development of the economy and society, particularly the increased pressure on the Social Medical Insurance Fund. Governments are actively engaged in public health education to alleviate problems associated with health risks (69). The core goal of health education is improving people's health by changing their health awareness and health behaviors (70). Public health education affects people in several ways. According to previous theories, public health education can improve people's health literacy, increase their health risk perceptions, and influence their health risk attitude, all of which may further promote the management of health risks through the purchase of commercial health insurance.

We focus on a vital question in this study: can public health education influence people's demand for commercial health insurance? Our research finds that the level of public health education significantly increases the density of commercial health insurance in a region, and this facilitation effect remains significant after overcoming endogeneity bias and conducting robustness tests. As the relationship between public health education and residents' demand for commercial health insurance remains underexplored, this original study can address this gap.

Furthermore, we uncover the “black box” of public health education to promote the purchase of commercial health insurance. We confirmed that health education can promote the purchase of commercial health insurance through three mediating variables: health literacy, health risk perception, and health risk attitudes (12, 28, 39). Through public health education, the health literacy of residents is improved, therefore, they may actively seek medical care (71, 72). Consequently, increased pressure on healthcare spending significantly promotes risk diversification through the purchase of commercial health insurance (9, 10, 21, 22). Due to the implementation of public health education, resident's health risk awareness has increased (22, 24). Consequently, they are more likely to purchase commercial health insurance to actively manage their health risks (23, 32). Through continued implementation of public health education, residents may become more averse to the risk of diseases, and more inclined to purchase insurance (7, 33, 36).

We also find significant differences in the impact of public health education on the development of commercial health insurance among regions with different demographic and socioeconomic characteristics. In terms of demographic characteristics, the impact of public health education on regional commercial health insurance development is greater in regions with a higher proportion of men, level of education, and urbanization rate. Men tend to prefer risks, and may have a greater change in risk attitude and manage health risks through commercial health insurance after receiving public health education (53). In areas with high education levels, residents are more likely to participate in health education and gain a better understanding about it, which leads them to actively manage their health risks by purchasing commercial health insurance (73). Additionally, a social welfare gap exists between urban and rural areas in China, which can affect the level of residents' participation in health education (74, 75). Rural residents are generally less aware of health education, which can further affect the development of commercial health insurance (45, 63). In terms of socioeconomic development, the promotion of health education for commercial health insurance is more evident in regions with high levels of economic development (75–77), abundant medical resources, and high social medical insurance coverage. After receiving health education, people with high income levels have more willingness and purchasing power to diversify their health risks by purchasing commercial health insurance (50, 51). In areas with abundant medical resources, people are more willing to purchase commercial health insurance to diversify the risk of medical costs because of the high accessibility of medical resources and willingness to seek medical care (53, 60). In areas with high rates of social medical insurance coverage, people have stronger health insurance awareness and cognition; therefore, they are more willing to purchase commercial health insurance (20).

## Limitations and future directions

This study has some limitations. First, this study examines the impact of public health education on residents' demand for commercial health insurance. However, our data resources do not provide individual data of public health education and commercial health insurance demand. Hence, we used provincial data instead. Second, commercial health insurance includes various types, such as medical insurance, long-term care insurance and major illness insurance. Each type of insurance has its own specific functions. However, we could only study the impact of public health education on the demand for residents' commercial health insurance from the overall level. In the future, we can further obtain data at the micro level through questionnaires and other tools and use it for further research. We may also concentrate on the impact of public health education on demand for different types of commercial health insurance. Additionally, previous studies have found different effects for different forms of public health education. Therefore, we can also study the effects of different forms of public health education on residents' demand for commercial health insurance in future.

## Conclusions and policy implications

Using panel data from the 2009–2019 period for 31 provinces (including municipalities and autonomous regions) in China, our study found that public health education can promote people's demand for commercial health insurance using a fixed effects model. This conclusion remains relevant after solving the endogeneity problem and conducting the robustness tests. Further, by conducting a mechanism analysis, we found that public health education can increase people's demand for commercial health insurance in three ways: improving health literacy and health risk perceptions, and changing health risk attitudes. Through heterogeneity analysis, we found that the effect of health education on promoting people's demand for commercial health insurance is more obvious in regions with high levels of urbanization, proportion of men, education, economic development, medical resources, and social medical insurance coverage.

Our findings provide important theoretical support and empirical evidence for countries managing residents' health risks through public health education. Countries should focus on health education and establish a set of scientific and complete public health education programs based on the disease spectrum. Additionally, they can improve the effectiveness of public health education implementation by enriching public health education modes, improving personalization, and increasing relevant financial input.

Second, countries are supposed to improve the quality of commercial health insurance. Improvements need to be made in the insurance industry by exploring new products and services, optimizing the structure of health insurance products, appropriately reducing health insurance rates, and improving the security of health insurance. High-quality development of the health insurance industry is significant in public health education for improving people's health risk protection.

**Figure 6:**
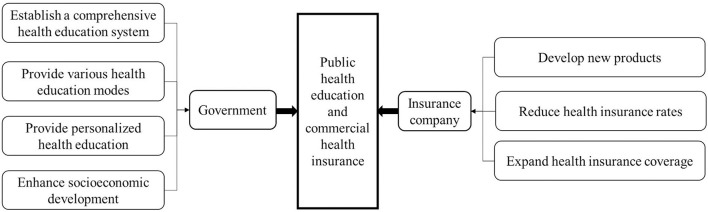
Measures to improve public health education and commercial health insurance.

Finally, countries should take various measures to improve the level of health risk protection for residents. This study found that the effect of public health education on people's demand for commercial health insurance is dependent on many factors, including demographic socioeconomic characteristics. Therefore, governments should continuously improve the education level of the population, further improve the urbanization level, promote sustainable and healthy economic development, increase the input of health funds, and enrich medical resources in all provinces. And Figure 6 shows measures that can be taken by government and insurance companies. These measures can comprehensively improve the effect of public health education to promote the purchase of commercial health insurance and effectively improve the level of residents' health risk protection.

## Data availability statement

The original contributions presented in the study are included in the article/supplementary material, further inquiries can be directed to the corresponding author.

## Author contributions

LG conceived the conception and design of this research. LG and YN carried out the preparation of writing-original draft, including data extraction, and statistical analysis. GW funded this research. All authors read, revised, and approved the submitted manuscript.

## Funding

This work was supported by the Research Foundation for Youth Scholars of Beijing Technology and Business University, Academic Research Projects of Beijing Union University (number SK90202101), Major Program Project of the National Social Science Foundation of China under Grant (Nos. 13&ZD042 and 17ZDA090).

## Conflict of interest

Author FL was employed by China Life Reinsurance Company Ltd. The remaining authors declare that the research was conducted in the absence of any commercial or financial relationships that could be construed as a potential conflict of interest.

## Publisher's note

All claims expressed in this article are solely those of the authors and do not necessarily represent those of their affiliated organizations, or those of the publisher, the editors and the reviewers. Any product that may be evaluated in this article, or claim that may be made by its manufacturer, is not guaranteed or endorsed by the publisher.
